# Investigation of electrical and magnetic properties of ferro-nanofluid on transformers

**DOI:** 10.1186/1556-276X-6-264

**Published:** 2011-03-28

**Authors:** Tsung-Han Tsai, Ping-Hei Chen, Da-Sheng Lee, Chin-Ting Yang

**Affiliations:** 1 Department of Mechanical Engineering, National Taiwan University, No. 1, Sec. 4, Roosevelt Rd., Taipei 10617, Taiwan; 2 Department of Energy and Refrigerating Air-conditioning Engineering, National Taipei University of Technology, No. 1, Sec. 3, Chung-hsiao E. Rd., Taipei 10608, Taiwan; 3 Department of Mechanical and Computer-Aided Engineering, St. John's University, No. 499, Sec. 4, Tam-king Rd., Tamsui, Taipei 25135, Taiwan

## Abstract

This study investigated a simple model of transformers that have liquid magnetic cores with different concentrations of ferro-nanofluids. The simple model was built on a capillary by enamel-insulated wires and with ferro-nanofluid loaded in the capillary. The ferro-nanofluid was fabricated by a chemical co-precipitation method. The performances of the transformers with either air core or ferro-nanofluid at different concentrations of nanoparticles of 0.25, 0.5, 0.75, and 1 M were measured and simulated at frequencies ranging from 100 kHz to 100 MHz. The experimental results indicated that the inductance and coupling coefficient of coils grew with the increment of the ferro-nanofluid concentration. The presence of ferro-nanofluid increased resistance, yielding to the decrement of the quality factor, owing to the phase lag between the external magnetic field and the magnetization of the material.

## Introduction

In coming decades, new generations of electronic products such as mobile phones, notebooks, and e-paper will be developed with the primary goals of mobilization and miniaturization. New CMOS fabrication technology will be applied to fabricate the miniaturized IC of electronic products on silicon substrates, including on-chip micro-transformers. Several issues of on-chip micro-transformers have been investigated for many years [1-21]. Some researches focused on the material of the magnetic core [1-10] and the geometry of the transformer [11-14]. Some papers discussed the parasitic effect of the conductive substrates. Transformer losses become dramatic at high frequencies and limit the performance of the transformers. Previous studies have discussed in detail the causes of transformer losses such as parasitic capacitance, ohmic loss, and substrate loss [15-18]. Core loss from the solid magnetic core significantly affected the performance of the transformers. The solutions for the solid magnetic core loss were proposed [19-21].

Consequently, only a few studies addressed transformers with liquid magnetic cores. The liquid magnetic core, ferro-nanofluid, with its distinguishing features of low electric conductivity and super-paramagnetism is regarded as a solution to the core losses of eddy current and hysteresis. In this study, a ferro-nanofluid was applied as a liquid magnetic core in a transformer. The performance of the transformer with the ferro-nanofluids was measured, simulated, and compared with that of a transformer with an air core.

## Experiment

The ingredients of ferro-nanofluid used in this study were Fe_3_O_4 _nanoparticles, oleic acid, and diesel oil. The oil-based Fe_3_O_4 _nanofluid was synthesized by co-precipitation, surface modification, nanoparticles dispersing, and base-fluid phase changing [10].

**Figure 1:**
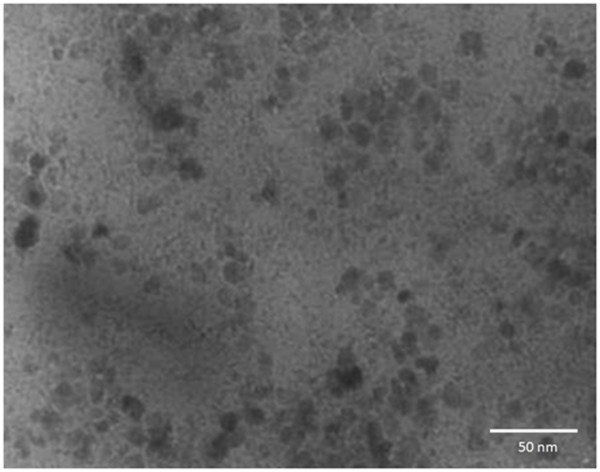
**The TEM photo of Fe_3_O_4 _nanoparticles**.

**Figure 2:**
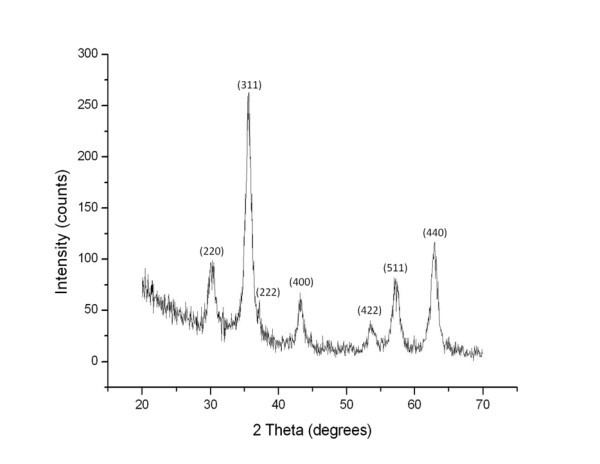
**The crystalline phases of Fe_3_O_4 _nanoparticles**.

**Figure 3:**
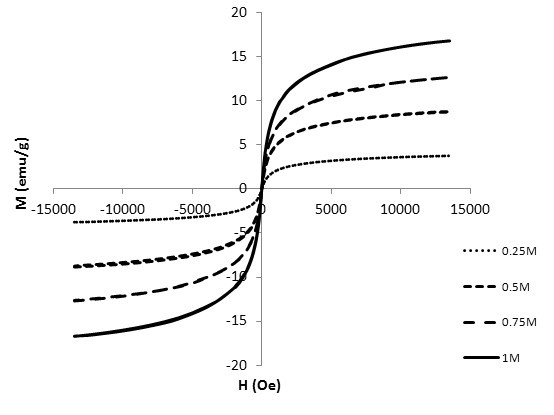
**The magnetized curve of the Fe_3_O_4 _nanofluid measured by a VSM**.

The shape and size of the Fe_3_O_4 _nanoparticles was examined by a transmission electron microscope (TEM). Figure 1 shows the TEM photo of the Fe_3_O_4 _nanoparticles. The average diameter of the nanoparticles was approximately 10 nm. The crystalline phases of Fe_3_O_4 _nanoparticles were determined by X-ray diffraction, as shown in Figure 2. The magnetic properties of Fe_3_O_4 _nanofluid were measured by a vibrating sample magnetometer (VSM). The magnetized curve of the Fe_3_O_4 _nanofluid measured by a VSM is shown in Figure 3. The measured results illustrate that the synthesized ferro-nanofluids have the characteristic of super-paramagnetism. The saturated magnetizations of 0.25, 0.5, 0.75, and 1 M Fe_3_O_4 _nanofluids were 3.75, 8.85, 12.7, and 16.7 emu/g, respectively.

**Figure 4:**
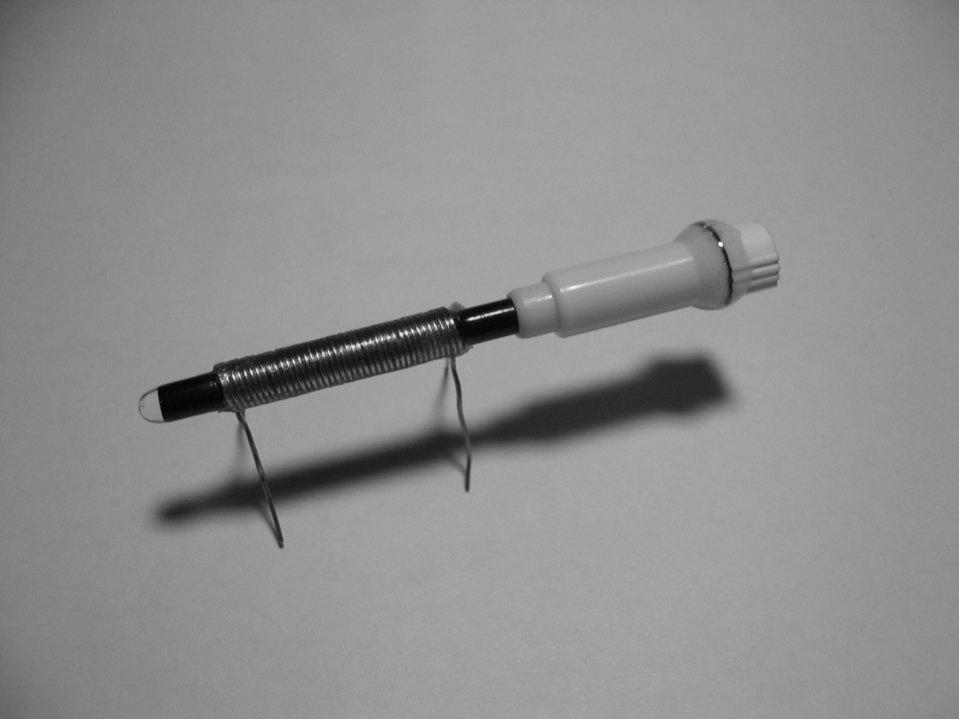
**The transformer on a capillary that loads the oil-based Fe_3_O_4 _nanofluid**.

A liquid magnetic core of a transformer was used in this study; the capillary served as a container in which the Fe_3_O_4 _nanofluid was loaded. The coils of the transformer were made by winding enamel-insulated wires on a capillary. Figure 4 shows the transformer on a capillary, which loads the oil-based Fe_3_O_4 _nanofluid. The diameter of the enamel-insulated wire used was 0.45 mm, and the thickness of the enamel layer was approximately 0.05 mm. The primary and secondary windings had 20 turns. The outer and inner diameters of the capillary were 3.2 and 2.3 mm, respectively, and the capacity of the capillary was 100 μL.

## Results and discussion

Different magnetic cores, air, and Fe_3_O_4 _nanofluids of 0.25, 0.5, 0.75, and 1 M were applied as the magnetic core of transformers. The inductance (*L*), coupling coefficient (*K*), resistance (*R*), and quality factor (*Q*) were measured by an Agilent 4294A Precision Impedance Analyzer. In this study, the simulation of the transformer was also established with HFSS 3D Full-wave Electromagnetic Field Simulation. By applying measured permeability, permittivity, and magnetic tangent loss and setting exciting sources, the impedances will be calculated by the finite element method. Both the frequencies of measurement and simulation range from 100 kHz to 100 MHz.

**Figure 5:**
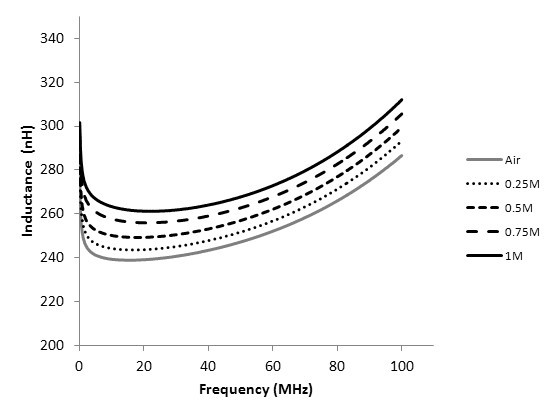
**The inductances of coils of transformers with different magnetic cores**.

**Figure 6:**
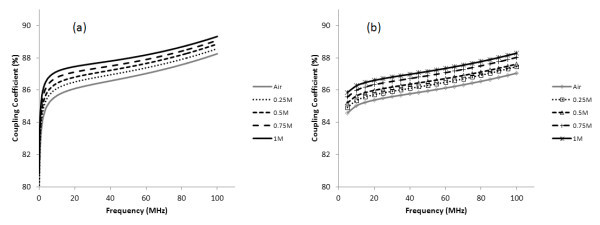
**The coupling coefficients of transformers with different magnetic cores**: **(a) **measured data; **(b) **simulated data.

Figure 5 shows the inductances of the coils of the transformers with different magnetic cores. Figure 5 illustrates that the inductance grows linearly with the increase of Fe_3_O_4 _concentration. At frequencies ranging from 100 kHz to 15 MHz, the inductances decrease rapidly due to the skin effect of coils. At frequencies ranging from 15 to 100 MHz, the inductances increase gradually and approach the maximum inductance at the resonance frequency. Figure 6 shows the measured and simulated results of the coupling coefficients of the transformers with different magnetic cores. The coupling coefficients also increase with the increase of Fe_3_O_4 _concentration. It increases rapidly below frequencies of 5 MHz and increases gradually with frequencies over 5 MHz. These results show that the magnetic cores of nanofluids can improve the inductance and coupling coefficients.

**Figure 7:**
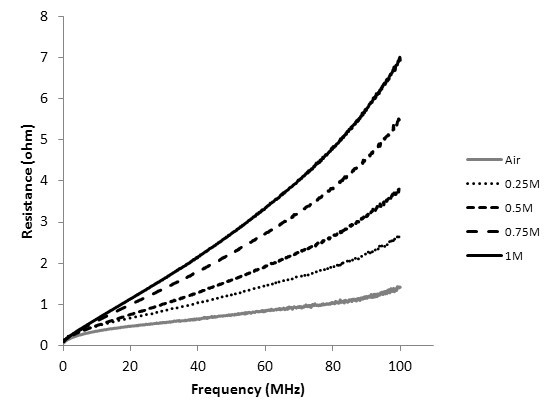
**The resistances of coils of transformers with different magnetic cores**.

Figure 7 shows that the resistance increases with the increase of Fe_3_O_4 _concentration, and it increases as a function of frequency. At 100 MHz, the resistances with the magnetic core of 0.25 and 1 M Fe_3_O_4 _nanofluids were two and five times the resistance as the air core. It is speculated that this is because of the phase lag on the material magnetization behind the external magnetic field at high frequencies. When the relaxation times cannot keep up the alternate time of the magnetic field, the resistance of the coils will grow rapidly [10,22]. At high frequencies, the permeability should be regarded as a complex number. Rearranging complex permeability and the inductance of a solenoid-type inductor, the impedance equation is obtained as follows:(1)

where ω is the angular frequency, *N *is the turns of coil, *A *is the cross-sectional area of solenoid, and *l *is the length of solenoid, μ" is the real part of complex permeability, and μ" is the imaginary part of complex permeability. It can be observed that the imaginary part of complex permeability μ" reflects on the real part of impedance, which is the cause of increasing resistance. Then, the quality factor *Q*, which is defined as the ratio of inductance to resistance, becomes [10]:(2)

**Figure 8:**
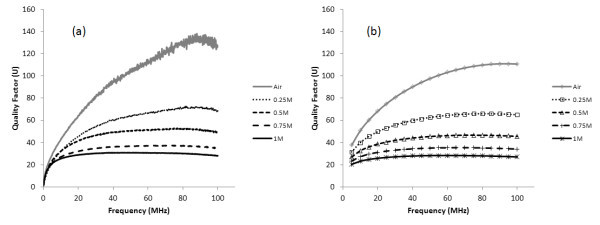
**The quality factors of coils of transformers with different magnetic cores**: **(a) **measured data; **(b) **simulated data.

Figure 8 shows the quality factor of coils of transformers with different magnetic cores. Owing to the fact that the increase of resistance is larger and faster than that of inductance with the presence of Fe_3_O_4 _nanofluids, the quality factor decreases when the Fe_3_O_4 _concentration rises. The simulated results show the same trend.

## Conclusions

In this study, different concentrations of ferro-nanofluids were applied to the magnetic cores of transformers. The performance of transformers with magnetic cores of air and Fe_3_O_4 _nanofluids of 0.25, 0.5, 0.75, and 1 M were measured, simulated, and compared. The experimental results indicated that the presence of Fe_3_O_4 _improved the inductance and the coupling coefficient of the coils. Due to phase lag on the material magnetization behind the external magnetic field at high frequencies, the resistance increased larger and faster than inductance, thus yielding a lower quality factor. For a micro-transformer, if a solid magnetic core is needed for higher inductance, it could be achieved by adding ferro-nanofluid and removing the base fluid repeatedly. This method has a lower thermal budget than the processes that sputtered or electroplated materials on chips. It is compatible with the MEMS process.

## Abbreviations

TEM: transmission electron microscope; VSM: vibrating sample magnetometer.

## Competing interests

The authors declare that they have no competing interests.

## Authors' contributions

TH performed experimental investigations of electric and magnetic properties of ferro-nanofluids on transformers and prepared the draft, PH proposed the phenomena for investigation and revised the manuscript, DS suggested the theory for the explanation of measured results, and CT designed the experimental systems. All authors read and approved the final manuscript.

## References

[B1] Ryu HJ Han SH Kim HJ Characteristics of twin spiral type thin film inductor with Fe-based nanocrystalline core IEEE Trans Magn 1999 35 3568 3570 10.1109/20.800592

[B2] Kim CS Bae S Kim HJ Nam SE Kim HJ Fabrication of high frequency DC-DC converter using Ti/FeTaN film inductor IEEE Trans Magn 2001 37 2894 2896 10.1109/20.951339

[B3] Kim KH Kim J Kim HJ Han SH Kim HJ A megahertz switching DC/DC converter using FeBN thin film inductor IEEE Trans Magn 2002 38 3162 3164 10.1109/TMAG.2002.802401

[B4] Zhuang Y Rejaei B Boellaard E Vroubel M Burghartz JN Integrated solenoid inductors with patterned, sputter-deposited Cr/Fe10C090/Cr ferromagnetic cores IEEE Electron Dev Lett 2003 24 224 226 10.1109/LED.2003.810880

[B5] Brandon EJ Wesseling E White V Ramsey C Castillo LD Lieneweg U Fabrication and characterization of microinductors for distributed power converters IEEE Trans Magn 2003 39 2049 2056 10.1109/TMAG.2003.812705

[B6] Wang N O'Donnell T Roy S Brunet M McCloskey P O'Mathuna SC High-frequency micro-machined power inductors J Magn Magn Mater 2005 290-291 1347 1350 10.1016/j.jmmm.2004.11.434

[B7] Gao XY Cao Y Zhou Y Ding W Lei C Chen JA Fabrication of solenoid-type inductor with electroplated NiFe magnetic core J Magn Magn Mater 2006 305 207 211 10.1016/j.jmmm.2005.12.014

[B8] Lei C Zhou Y Gao XY Ding W Cao Y Choi H Won JH Fabrication of a solenoid-type inductor with Fe-based soft magnetic core J Magn Magn Mater 2007 308 284 288 10.1016/j.jmmm.2006.06.002

[B9] Lee DS Energy harvesting chip and the chip based power supply development for a wireless sensor network Sensors 2008 8 7690 7714 10.3390/s8127690 PMC3790984 27873953

[B10] Tsai TH Kuo LS Chen PH Lee DS Yang CT Applications of ferro-nanofluid on a micro-transformer Sensors 2010 10 8161 8172 10.3390/s100908161 PMC3231209 22163647

[B11] Prieto MJ Pernia AM Lopera JM Martin JA Nuno F Design and analysis of thick-film integrated inductors for power converters IEEE Trans Ind Appl 2002 38 543 552 10.1109/28.993177

[B12] Seemann K Leiste H Beckker V A new generation of CMOS-compatible high frequency micro-inductors with ferromagnetic cores: theory, fabrication and characterization J Magn Magn Mater 2006 302 321 326 10.1016/j.jmmm.2005.05.042

[B13] Yamaguchi M Kim KH Ikedaa S Soft magnetic materials application in the RF range J Magn Magn Mater 2006 304 208 213 10.1016/j.jmmm.2006.02.143

[B14] Dai CL Chen YL Modeling and manufacturing of micromechanical RF switch with inductors Sensors 2007 7 2660 2670 10.3390/s7112670 PMC3965228 28903253

[B15] Yoon JB Kim BI Choi YS Yoon E 3-D construction of monolithic passive components for RF and microwave ICs using thick-metal surface micromachining technology IEEE Trans Microw Theory Techn 2003 51 279 288 10.1109/TMTT.2002.806511

[B16] Chong K Xie YH High-performance on-chip transformers IEEE Electron Dev Lett 2005 26 557 559 10.1109/LED.2005.851817

[B17] Yunas J Hamzah AA Majlis BY Fabrication and characterization of surface micromachined stacked transformer on glass substrate Microelectron Eng 2009 86 2020 2025 10.1016/j.mee.2008.12.091

[B18] Yunas J Hamzah AA Majlis BY Surface micromachined on-chip transformer fabricated on glass substrate Microsyst Technol 2009 15 547 552 10.1007/s00542-008-0704-2

[B19] Xu M Liakopoulos TM Ahn CH A microfabricated transformer for high-frequency power or signal conversion IEEE Trans Magn 1998 34 1369 1371 10.1109/20.706551

[B20] Park JW Allen MG Ultralow-profile micromachined power inductors with highly laminated Ni/Fe cores: application to low-megahertz DC-DC converters IEEE Trans Magn 2003 39 3184 3186 10.1109/TMAG.2003.816051

[B21] Zhao JH Zhu J Chen ZM Liu ZW Radio-frequency planar integrated inductor with permalloy-Si02 granular films IEEE Trans Magn 2005 41 2334 2338 10.1109/TMAG.2005.852949

[B22] Kotitz R Weitschies W Trahms L Semmler W Investigation of Brownian and Neel relaxation in magnetic fluids J Magn Magn Mater 1999 201 102 104 10.1016/S0304-8853(99)00065-7

